# Neuroimaging of neonatal brain post therapeutic hypothermia: a practical guide to the non-pediatric neuroradiologist

**DOI:** 10.3389/fradi.2026.1722473

**Published:** 2026-02-17

**Authors:** Martina Di Stasi, Angela Plantulli, Felicia Filomena Varsalone, Gianpiero Locatelli, Chiara Paolella, Giulia Frauenfelder, Margherita Di Stasi, Francesco Taglialatela, Maria Grazia Corbo, Daniele Giuseppe Romano

**Affiliations:** 1 Department of Diagnostic and Interventional Neuroradiology, Azienda Ospedaliera Universitaria San Giovanni di Dio e Ruggi D'Aragona, Salerno, Italy; 2 Department of Pediatric Neurosciences, Neuroradiology, Azienda Ospedaliera di Rilievo Nazionale Santobono Pausilipon, Naples, Italy; 3 Ophthalmology Unit, Presidio Ospedaliero San Luca, Vallo Della Lucania, Italy

**Keywords:** hypoxic ischemic encephalopathy (HIE), magnetic resonance imaging (MRI), neonatal brain, non-pediatric neuroradiologist, therapeutic hypothermia (TH)

## Abstract

Therapeutic hypothermia is currently considered the standard treatment for neonates diagnosed with moderate or severe hypoxic ischemic encephalopathy in high-resource settings, improving survival rates and reducing long-term disability. Consequently, this treatment is increasingly performed in non-pediatric hospitals with intensive neonatal care units. Magnetic resonance imaging plays a fundamental role in assessing the extent of brain injury and represents a key prognostic tool in these patients who present to the neuroradiologist with critical care condition. As the current literature on this topic is flourishing, in this study, we aim to provide a practical guide to the non-pediatric neuroradiologist by summarizing protocols, characteristic radiological findings, and recommendations for ensuring optimal imaging timing by revising published studies.

## 1 Introduction

Neonatal hypoxic ischemic encephalopathy (HIE) is a major cause of long-term disability and death worldwide, with a prevalence of 1–4/1,000 live births in developed countries rising to as high as 26/1,000 live births in low- and middle-income countries (1).

HIE is a complex and evolving pathological process triggered by prenatal, intrapartum, or postnatal events, leading to impaired brain perfusion and oxygen supply, resulting in a primary failure of oxidative metabolism with ATP depletion, cytotoxic cell edema, and release of excitatory amino acids. These excitotoxins contribute to the loss of cellular integrity through receptor binding and subsequent influx of electrolytes as calcium and sodium, ultimately leading to apoptosis and necrosis. During the second phase, restoration of cerebral blood flow (CBF) promotes excessive free radical production, further inflammation, and microglial activation, thereby exacerbating cerebral damage (2). Since its approval by the United States Food and Drug Administration in 2007, therapeutic hypothermia (TH) rapidly became the emerging treatment for neonates with moderate to severe HIE in high-income countries (HICs) (3), although evidence remains less robust in low- and middle-income settings (4), representing one of the major improvements in neonatal care. Several randomized controlled trials demonstrated that the cooling process, when initiated within 6 h of birth, reduces mortality at 18–24 months in HICs and decreases the incidence of cerebral palsy and adverse neurodevelopmental outcomes in both high- and low-resource settings (5–8). The exact mechanism by which TH exerts neuroprotective effects is not fully understood, but cooling seems to reduce cerebral metabolism by approximately 5% for each degree of temperature decrease, with a consequent reduction of glutamate, nitric oxide, and free radical release (9, 10). Nevertheless, approximately 30% of cooled neonates still develop adverse neurological sequelae despite treatment (5). This may be partly explained by the selective regional efficacy of hypothermia, as several authors have hypothesized that specific brain regions such as the cortex and deep gray nuclei may experience greater neuroprotection than parasagittal regions (11). In this regard, HIE is notoriously associated with specific brain damage patterns, well characterized by magnetic resonance imaging (MRI), which currently represents an indispensable tool to define the extent of brain injury and helps in short- and long-term outcome prediction for both cooled and non-treated neonates with HIE (12). Currently, TH has been successfully translated to routine practice and is performed in many non-pediatric hospitals with a neonatal intensive care unit (NICU): consequently, non-dedicated neuroradiologists are increasingly dealing with complex cases, whose interpretation might be influenced by the effects of cooling itself (13). The Newborn Brain Society (NBS) and the Canadian Pediatric Society (CPS) strongly recommend that MR images should be interpreted by a trained neuroradiologist with neonatal and pediatric expertise; when no dedicated physician is available on site, images should be referred to a specialized structure for evaluation (12, 14). Unfortunately, this is not always achievable, and at the same time, although various guidelines have been developed by different authors, there is no international standardized MRI protocol for HIE neonates treated with TH (12). Given these reasons, and as the literature on this topic is flourishing, in this study, we aim to provide a practical guide to the non-pediatric neuroradiologist by summarizing MR protocols, recommendations for ensuring imaging timing, and interpretation by reviewing published works.

## 2 Literature review

We reviewed the existing literature by searching on Scopus (https://www.scopus.com), PubMed (https://pubmed.ncbi.nlm.nih.gov), and Google Scholar (https://scholar.google.com) using the following keywords: [“therapeutic hypothermia” AND/OR “perinatal asphyxia” AND/OR “neonatal hypoxic ischemic encephalopathy” AND/OR “neonatal cerebral injury” AND [“neuroimaging” AND/OR “MRI” AND/OR “MRI score”].

We initially included articles published between January 2000 and September 2025 and subsequently screened the reference lists of selected papers to include further relevant or landmark scientific studies. Finally, additional articles were included as needed to ensure the comprehensiveness of the review. Two neuroradiologists with 15 (GL) and 8 (MDS) years of experience, respectively, in pediatric imaging independently screened titles and abstracts and evaluated the papers with regard to the reported specific neuroimaging findings, MR timing, dedicated MR sequences, their diagnostic value for neonatal brain injury, neurological outcomes, clinical indications, and MRI prognostic scoring systems. Case reports or case series, letters to the Editor, conference abstracts, and non-English language articles were excluded. This approach resulted in a rigorous (though not systematic) survey appropriate for a mini-review.

## 3 Clinical HIE

Eligibility criteria for TH include gestational age >35 weeks, birth weight >1,800 g, postnatal age <6 h, and evidence of peripartum asphyxia based on clinical criteria such as an APGAR score <5 at 10 min of life, prolonged resuscitation at birth, umbilical cord pH <7.0 or a base deficit >12 mmol/L within 60 min of life, or the presence of moderate to severe encephalopathy diagnosed by the neonatologist using the Sarnat staging system. A pathological amplitude-integrated electroencephalography (aEEG) pattern also constitutes an indication for TH (15–20): several studies have demonstrated its association with MRI-detected lesions in the basal ganglia and thalamic (BGT) region, white matter, and the posterior limb of the internal capsule (PLIC) (21). Cooling is typically initiated within the first 6 h after birth. The neonate's temperature is lowered to 33 °C–34 °C using servocontrolled devices and maintained for 72 h. During the rewarming phase, temperature is gradually increased by approximately 0.5° per hour until a target temperature of 36.5 °C is reached (22). Continuous monitoring of vital signs such as heart and respiratory rate, oxygen saturation, and electrolyte balance is mandatory throughout the procedure. Whenever possible, aEEG monitoring should be performed before and during cooling to detect potential seizures and allow prompt initiation of anticonvulsant treatment. A placental histopathological analysis is commonly performed to identify the underlying pathologies. At the time of NICU discharge, healthcare providers should inform the parents of neonates about the prognosis made and guide the family to undergo appropriate neurodevelopmental follow-up. Current recommendations suggest follow-up of these patients until at least 24 months of age and, when feasible, through the preschool years (23).

## 4 Imaging

Cranial ultrasound (cUS) is commonly used as a first-line imaging modality in neonates and can be useful for excluding intracranial hemorrhage, which constitutes a contraindication to TH, or major congenital malformations (24). Nevertheless, cUS has limited sensibility in distinguishing ischemic from hemorrhagic lesions, detecting punctate white matter lesions (PWMLs) and cerebellar injury, and assessing cortical abnormalities (25). MRI remains the gold standard to delineate brain damage in HIE neonates.

### 4.1 MRI protocols

As previously noted, no internationally standardized MRI protocol currently exists for neonatal HIE. Neonatal brain MRI is typically performed using 1.5 or 3T scanners, with adequate imaging quality in both cases. When feasible, a 3T scanner should be preferred because of its higher signal-to-noise ratio (SNR), which can be leveraged to achieve higher resolution or thinner sections without increasing the scanning time (26). Because of the limited availability of dedicated neonatal head coils, which improve signal quality (27), many centers rely on adult 32 or 12-channel head coils (28, 29).

Neonatal brain imaging is technically challenging, as MR sequences must be tailored to the small head size, tissues composition, incomplete myelination, and ongoing brain maturation, all of which result in signal characteristics distinct from those of the adult brain. For clinical purposes, the MRI protocol should include at least five conventional sequences (30):

- 3D gradient-echo (GrE) T1w or 2D axial T1w images: 3D imaging is preferable for obtaining detailed anatomic information, but good-quality 2D axial inversion recovery (IR) T1w is also suitable for assessing myelination and detecting permanent brain damage; visible as abnormal T1w hyperintensity (27).
- 2D axial and coronal turbo spin echo (TSE) T2w images: These are useful for defining anatomy, myelination, and white matter signal abnormalities such as cerebral edema visible as T2w hypersignal (31).
- Diffusion-weighted imaging (DWI) with apparent diffusion coefficient (ADC) maps: Acute injury appears as areas of parenchymal signal abnormality associated with diffusion restriction, observable within the first hours following the pathological event, when conventional images are still unremarkable (32). These changes are most pronounced at 2–4 days after birth.
- Susceptibility weighted imaging (axial GrE T2*w, BOLD, or SWI sequences): These are usually used to reveal intraparenchymal, intraventricular, and extra-axial hemorrhages or calcifications. In addition, SWI has been proven to show abnormal cerebral venous signals due to reduced blood flow and perfusion with consequent deoxygenation in HIE neonates (33).

**Figure 1:**
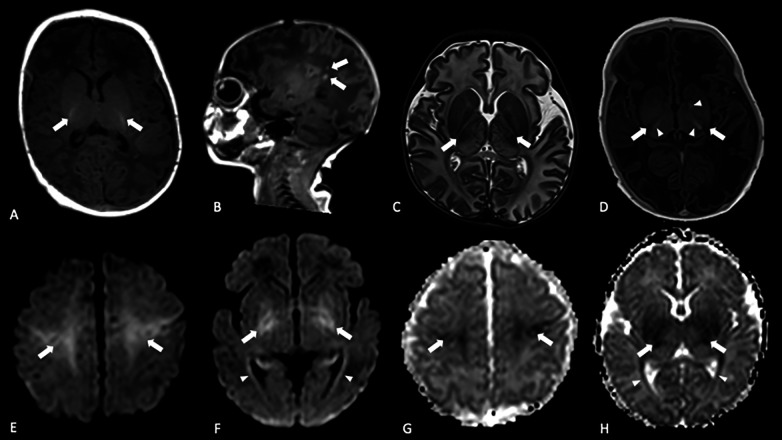
Brain MRI in term HIE neonates treated with TH: **(A)** axial 3D GrE T1w showing the physiological PLIC hyperintensity (arrows); **(B)** sagittal 3D GrE T1w demonstrating focal areas of high signal due to PWMLs (arrow);**(C)** axial TSE T2w showing the absence of the typical PLIC hypointensity (arrows); also, note the abnormal thalamic signal; **(D)** axial IR T1w showing the loss of the typical PLIC hyperintensity (arrows) and exaggerated hypersignal in the thalami and left globus pallidus (arrowheads); **(E,F)** axial DWI and **(G,H)** the corresponding ADC maps showing restricted diffusion in the perirolandic cortex, BGT regions (arrows), and along optic radiation (arrowheads).

Normal myelination in the newborn is better appreciated on T1w images, appearing as a spontaneous hypersignal within the PLIC at approximately 36–37 weeks of gestation (Figure 1A). A similar pattern should be noticed in thalamic and subthalamic nuclei, perirolandic areas, and the dorsal brainstem (31); PLICs are often injured in HIE, and the loss of their normal signal intensity on T1 and T2w images is predictive of severe adverse motor outcomes (34). While DWI lesions can be evident a few hours after birth and persist until pseudonormalization, conventional T1w and T2w sequences can initially show only subtle alterations, which become more evident 3–4 days later. A careful interpretation of diffusion-weighted images is essential as DWI changes are not exclusively attributable to hypoxic ischemic etiology but may also occur in the event of hypoglycemia, seizures, and metabolic alterations (35).

Additional sequences can be performed if the patient's clinical condition allows the following:

- Vascular sequences such as 3D MR venography (MRV) and arteriography (MRA) are used to exclude neonatal stroke and cerebral sinovenous thrombosis (31).
- Magnetic resonance spectroscopy (MRS): It allows the study of neurometabolite concentrations in brain tissue, but it also involves significant technical challenges because of motion artifacts, decreased shimming due to the small brain size, and different T_1_/T_2_ relaxation times compared with adult brain tissue (36). Different authors have recommended the addition of H-MRS to the neonatal MRI protocol because an increased lactate/N-acetyl-aspartate (NAA) ratio seems to be a predictor of unfavorable outcomes (37, 38). The chemical shift imaging technique with a long echo time and monovoxel study is preferable to the multivoxel method because of reduced artifacts and improved spectrum quality. When MRS is performed with an echo time (TE) of 144 ms, a lactate peak can be easily identified as an inverted doublet at 1.33 ppm. A decrease in NAA levels can also be detected. The volume of interest is usually placed at the level of the basal ganglia, avoiding cerebrospinal fluid and white matter (39). When evaluating the H-MRS spectrum, the neuroradiologist has to consider that in the neonatal brain, the metabolic profile changes in respect of brain maturation and different brain regions, and that the choline peak is physiologically larger than that of NAA due to the rapid growth and turnover of the membranes (40).
- Arterial spin labeling (ASL) perfusion imaging: This non-contrast sequence uses endogenous water from the blood as a perfusion tracer to obtain CBF maps, which can be helpful in the evaluation of brain blood flow redistribution (41).

Wintermark et al. demonstrated that an abnormal CBF in the first days of life (DOL) could identify areas of future injury on the subsequent MR images in both cooled and untreated HIE neonates, and that despite TH, the detection of hypoperfusion changes with the successive onset of hyperperfusion patterns could be a predictor of brain damage (28). However, it should be noted that ASL images can be affected by sedation.

Further sequences as diffusor tensor imaging (DTI) and functional MRI (fMRI) are usually performed for research aims and fall outside the purpose of this review; it is obligatory, however, to know that certain DTI parameters such as fractional anisotropy (FA) and ADC are promising prognostic tools (42) and that fMRI in neonates with severe HIE showed brain function network underdevelopment (43). Finally, recent improvements in radiomics and machine learning have enabled the development of high-accuracy models that predict neurodevelopmental outcomes in neonates with HIE by extracting quantitative MRI features beyond human visual assessment. These systems are still limited to research purposes but lead the way to innovative, automated methodologies (44). Advanced MRI techniques may detect alterations not visible on conventional imaging that could reveal subtle neurological impairments emerging later in life. Evidence shows that children with HIE perform worse than controls at school age across multiple domains, even in the absence of cerebral palsy or other clinically apparent neurological disabilities. Early identification of individuals who may develop motor, cognitive, or learning difficulties—despite lacking definite injury on conventional brain MRI—could enable timely access to adjunctive or synergistic interventions aimed at improving long-term outcomes (45).

Main imaging findings are reported for each sequence in Table 1.

**Table 1:** Main imaging findings, the corresponding timings, and the related pearls and pitfalls for each MR sequence.

Sequence	Timing	Imaging findings	Pearls and pitfalls
T1W	Shows only subtle alterations during the first hours after the acute event, which become evident within 3–4 days.	Both T1 shortening and elongation may indicate tissue damage. Typical findings include cortical highlighting and hypersignal in the rolandic cortex and BGT region, or the loss of physiological PLIC T1w hyperintensity. PWMLs also appear as small foci of T1w hyperintensity.	In healthy term neonates, PLIC shows higher signal intensity compared with posterolateral putamen and ventrolateral nucleus of thalamus. However, subtle and scarcer hyperintensity can also be seen in these structures, corresponding to normal myelination (79).
T2W	Shows only subtle alterations during the first hours after the insult, which become evident within 3–4 days.	Increased T2w signal may indicate edema. Loss of gray-white matter differentiation is best appreciated on T2w images. Inversion of the normal PLIC signal (T2w hypointensity and T1w hyperintensity) is a negative prognostic factor. PWMLs usually appear hypointense on T2w images.	White matter edema can be difficult to detect in newborns because of intrinsically high T2 values of the largely unmyelinated neonatal brain. Vasogenic edema becomes more conspicuous by Day 3 (80).
DWI/ADC	Becomes positive within the first hours after the acute event, reaching the peak within 2–4 days. Pseudonormalization occurs between 11 and 12 DOL in cooled neonates.	High signal on DWI with corresponding low ADC values indicates cytotoxic edema with specific distribution depending on the injury pattern. PWMLs can also show restricted diffusion. Patients with poor outcomes tend to exhibit lower ADC values in the basal ganglia and mesial thalamus (39).	Widespread diffusion restriction may be overlooked in the “near total injury” pattern because of the uniformity of signal change. If the cerebellum is spared, a comparison between supratentorial hyperintensity and normal infratentorial structures (the “white cerebrum sign”) can be helpful (81). Careful interpretation is essential, as restricted diffusion may also occur in seizures and metabolic disorders, and the timing of pseudonormalization depends on TH.
SWI/BOLD/T2*GrE	Detects both recent and chronic hemorrhage. Calcifications can be distinguished from blood products on filtered-phase images (82).	Susceptibility sequences are sensitive to distortion to local magnetic field distortion, with hemorrhagic foci appearing as areas of low signal. PWMLs in term HIE neonates are usually ischemic and therefore not visible on SWI (83). In some cases, prominent hypointense veins can be observed in injured areas because of increased compensatory oxygen extraction following hypoxia (34).	Enlarged deep medullary and cortical veins can be characteristically seen in HIE neonates in injured areas and in the presence of brain edema, supporting the diagnosis (84).
MRA/MRV	Useful for detecting thrombus in early PAIS. MRV easily assesses venous thrombosis.	Findings may include focal or segmental absence of the normal vascular flow signal or venous engorgement.	Hypoagenesia of dural venous sinuses should not be misinterpreted as thrombosis: when evaluating the segmental absence of typical flow signals, morphological sequences should also be analyzed. Signs of intracranial hypertension can support the finding.
ASL	Reveals perfusion abnormalities during the first week of life.	Early hypoperfusion may be noticed in PAIS. In HIE neonates a first hypoperfusion phase followed by hyperperfusion can be observed. Low CBF values may predict areas of subsequent injury and correlate with later abnormalities on conventional MR sequences (28).	ASL remains technically challenging in neonates because of lower CBF, related reduced SNR, contamination from large vessels, and a longer tracer lifetime. These factors require optimization of sequence parameters, for example reducing the delay between labeling and acquisition and the number of repetitions (85).
MRS	Metabolite alterations appear during the first days after birth and can persist for 1–2 weeks.	Decreased NAA/Cho ratio and increased lactate levels (inverted double peak at 1.33 ppm with TE 144 ms) are typical findings. These abnormalities predict unfavorable neurodevelopmental outcomes.	Lactate is naturally present in cerebrospinal fluid, so cerebrospinal spaces should be avoided when positioning the voxel of interest (86).

### 4.2 Neonates preparation

Several strategies can be implemented to achieve high-quality MRI examinations, and the first such strategy is a meticulous collaboration between NICU and MR staff. The MR site should be alerted prior to the neonatal transfer to allow the neuroradiology team to make all the necessary arrangements (46).

Achieving optimal immobility is crucial to obtain motion-artifact-free images.

The “feed and wrap technique” is utilized to induce natural sleep in neonates and consists of a first phase when the baby is fed in a room or protected area near the scanner and a second phase during which the patient is swaddled in a blanket. Dedicated vacuum immobilization devices, when available, should also be used (47).

Adequate hearing protection must be provided by using neonatal ear muffs.

The need for mild sedation should be evaluated on a case-by-case basis through discussions with the neonatologist.

Neonates with HIE undergoing brain MRI usually do not require intravenous contrast, making endonasal administration of sedative medications a suitable option to obtain non-deep sedation. Intranasal dexmedetomidine has been increasingly used in neonates, although it fails to ensure completely motion-artifacts-free images in approximately half of cases. Some authors have proposed the use of intranasal dexmedetomidine, together with midazolam, both for pediatric and for neonatal sedation, providing effective results and a good safety profile. The most frequent adverse effect (although reported in a small number of cases) is represented by desaturation, usually self-limiting or quickly responsive to oxygen supplementation. Caution should be exercised in case of renal or hepatic impairment with regard to drugs with a longer duration of action and during concurrent opioid administration. Last feeding is allowed for up to 2 h before the scheduled examination (48, 49).

Close monitoring of vital signs is mandatory during neonatal MRI, from the time of sedation until full waking, and these signs include heart rate, oxygen saturation, and respiratory rate. Extremely ill neonates could require adjunctive support for ventilation and drugs administration: as most infusion pumps are not MR-compatible, the dedicated team may place them near the magnet entrance and realize such connection using long tubing devices (46).

### 4.3 Major patterns of brain injury

The main MRI-defined brain injury patterns in neonates with HIE are central or basal ganglia (25%–75%), watershed (15%–45%), punctate white matter lesions (10%–20%), near total injury (5%–10%), brainstem (5%–20%), cerebellum (9%), and perinatal arterial ischemic stroke (PAIS) (<1%) (27). Importantly, it is essential to stress that multiple injury patterns may coexist in the same neonate.

The BGT pattern is typically associated with impaired placental perfusion or reduced umbilical oxygen delivery, resulting in profound hypoxia (34). Imaging findings include bilateral and usually symmetric signal alterations in the basal ganglia and thalami, with a predilection for ventrolateral nuclei of the thalamus, lentiform nuclei, and perirolandic cortex (Figures 1E–H). Hippocampi can also be involved, but damage is usually more difficult to detect in the acute phase (12). The typical T1w high signal usually seen in term neonates within the ventral–lateral nucleus of the thalamus and the posterior part of the lentiform nucleus can be exaggerated in case of HIE; the inverted signal intensity of the PLIC on T1- and T2-w (hyposignal on T1w and hypersignal on T2w) (Figures 1C,D) is highly predictive of severe adverse motor outcomes, as highlighted by several studies (50).

Segmental or focal abnormal cortical highlighting in the perirolandic region, with a shortening of both T1 and T2, is another characteristic finding (51).

The parasagittal (watershed) pattern is related to prolonged maternal hypotension, sepsis, or hypoglycemia, typically involving the cerebral cortex and contiguous white matter along vascular border zones between the major cerebral arteries territories, most commonly in the parieto-occipital lobes (12, 52). Imaging features comprise increased T2w signal in white matter, lack of differentiation between gray and white matter, and local brain swelling. DWI can show signs of restricted diffusion in injured areas (53).

PWMLs are more frequently observed in near-term neonates, representing focal areas of ischemia or necrosis. They are defined as spots (not larger than 5 mm) of T1w hypersignal (Figure 1B) and decreased T2w signal, sometimes showing restricted diffusion with a cluster, linear, or mixed-type pattern. Different from those appreciated in premature infants, PWMLs in term babies are often not visible on SWI images. White matter alterations have been associated with chorioamnionitis, impaired placental maturation, and genetic mutations (54).

The “near-total” injury pattern represents the most severe scenario with neuronal necrosis across the cortex, basal ganglia and thalami, midbrain and brainstem, and related diffuse signal abnormalities. Sometimes, the cerebellum may be spared (12, 55).

The brainstem pattern is associated with damage of the brainstem tegmentum, often with concomitant injury to the BGT: involvement of the inferior olivary nuclei is a characteristic feature (51).

The cerebellum is a structure more resistant to hypoxic–ischemic damage, but sometimes, areas of diffusion restriction and abnormal T1w signal have been recognized at the level of dentate nuclei and vermis (56).

PAIS frequently presents with neonatal encephalopathy and seizures. It is easily recognized because of its wedge-like shape and localization to a vascular distribution, with diffusion restriction, gray and white matter loss of differentiation, and sulcal effacement (12). DWI abnormalities can also extend within the corticospinal tracts, PLICs, and mesencephalon (pre-Wallerian degeneration) (57).

Injury to mammillary bodies (MBs) has long been overlooked as they constitute small structures best seen on thin (2–3 mm) images, and therefore, their damage can be easily missed, especially if they are localized with the brain otherwise spared. MBs are part of the Papez circuit, receiving inputs from hippocampi and projecting through mammillo-thalamic tracts to anterior thalamic nuclei. Acute damage can be seen as areas of T2w hyperintensity, swelling, and diffusion restriction, with consequential atrophy which is related to memory dysfunction and worse cognitive outcomes (58, 59).

### 4.4 Mimics and pitfalls

One of the most significant lessons a neuroradiologist must learn is that it is not always possible to distinguish a pathology from its mimickers blinded to clinical information. Close cooperation with the clinician is fundamental for accurate imaging interpretation: type of delivery, maternal diseases, symptom onset, and laboratory tests are essential elements for reaching the correct diagnosis.

Neurometabolic disorders are potential HIE mimickers, but usually neonates show a perinatal normal interval because of the support provided through the placenta before delivery (60). The most frequent types are listed below.

Urea cycle disorders and molybdenum cofactor -sulfite oxidase deficiency can affect the basal ganglia, cortex, and subcortical white matter with T1w and T2w elongation and/or DWI hypersignal. Subsequent T1w shortening in high myelinated structures can mimic the subacute HIE phase. An increased peak of glutamine/glutamate and a decrease in NAA and choline peaks can be encountered in the first and second cases, respectively (61–63). Maple syrup urine disease shows an inversion of the typical PLIC signal and T2w hyperintensity in subcortical white matter and a methyl peak may be seen at 0.9 ppm, while non-ketotic hyperglycinemia characteristically presents with restricted diffusion in PLICs, the anterior brainstem, the posterior tegmental tracts, and the cerebellum (64, 65). Glutaric aciduria can resemble the BGT pattern as it induces neurotoxicity at this level with associated macrocephaly and widened sylvian fissures. Elevated urinary excretion of glutaric acid can be easily assessed (66).

### 4.5 When to scan

The issue of optimal MRI timing has been extensively debated, and different authors have proposed diverse approaches as MRI findings may be influenced by postnatal age (19, 21, 67).

Early scans acquired before 4–5 DOL typically show acute damage: at this time, DWI is the best sensitive sequence well depicting primary injury. Conversely, delayed imaging performed at approximately10 days of life may better demonstrate the full extent of damage by relying on T1w and T2w changes, because DWI alone may not reflect all areas of irreversible cell death and necrosis (24, 35).

TH technical timing represents another key aspect: usually neonates with HIE are cooled for 72 h and then gradually rewarmed over 24 h; therefore, MRI cannot be performed during this period. To date, MRI acquisition during TH is generally limited to research purposes (27). Some studies have reported that delayed MRI may underestimate DWI lesions because of the occurrence of ADC pseudonormalization at 7–8 days of postnatal age. However, Bednarek et al. have recently demonstrated that DWI pseudonormalization appears approximately between 11 and 12 DOL in neonates treated with TH and approximately at 7 DOL in untreated HIE infants (13). This is a critical consideration for both imaging interpretation and scan timing definition.

The American College of Obstetricians and Gynecologists, the CPS, and the NBS recommend the performance of two MRI examinations: the first after cooling and the second at 10–14 DOL, especially when clinical symptoms are not consistent with abnormalities found on early scans (12, 14, 68).

Unfortunately, this is often not feasible, particularly in non-pediatric hospitals without dedicated MR sessions. The key may lie in achieving an appropriate balance between ease of acquisition and evaluation of full injury. Some authors have therefore proposed performing MRI at 10–14 DOL, when the extent of cerebral injury can be accurately assessed (24, 35, 68)*.* In practice, imaging at 9–10 DOL may represent a reasonable compromise, as ADC pseudonormalization is unlikely to have occurred, T1w and T2w changes should be clearly detectable, and the neonatal clinical conditions are generally stable.

### 4.6 Practical limitations and protocol variability

Significant variability exists in TH practice and MRI implementation at both global and regional levels. This inconsistency is largely attributable to variations in national guidelines, inclusion and exclusion criteria across published controlled trials, lack of care standardization during hypothermic treatment, and subsequent short and long-term follow-ups (3).

Major differences comprise use of various eligibility scores systems (e.g., Sarnat, Thompson, Siben, and Brigham and Women's Hospital (BWH) neonatal encephalopathy scores), leading to dissimilarities in HIE grading and patient selection, disparities in resource availability, use of aEEG before and during TH, gestational age cutoff (most centers offer TH at a gestational age ≥35 or ≥36, whereas some studies propose 34 weeks as the lower limit), and therapeutic window (69). The first neurodevelopmental follow-up is performed at 2–4 months in most sites, but some centers manage earlier assessments at 2–4 weeks, with the total follow-up duration ranging from 12–24 months to 3 years (3, 70). In addition, different clinical scales are used to evaluate neurodevelopment outcomes (with the Griffiths Scales of Child Development and Bayley Scales of Infant Development being the most commonly used).

MRI timing and protocols also vary considerably among centers, as discussed in previous paragraphs.

In many cooling centers, neuroradiologists are not exclusively pediatric-dedicated specialists but are required to interpret brain, spine, and peripheral nervous system imaging in all age groups; consequently, neonatal scan interpretation may demand additional effort, constant training, and regular updates.

These disparities may result in data inconsistency and reporting gaps, highlighting the urgent need for future international guidelines focusing on protocol harmonization, resource availability, cohesive strategy, and standardized reporting to optimize the effectiveness of TH and to define consistent outcome goals.

## 5 Scoring and outcome

Several MRI-based scoring systems have been developed to grade brain injury severity: most authors have utilized a modified Rutherford score, which evaluates anomalies in the basal ganglia, thalami, PLICs, corpus callosum, white and gray matter, brainstem, and cerebellum. Each area is assessed on four MR sequences (19, 21, 37, 71). The Barkovich score has also been reported in different studies: it assesses the basal ganglia (scored from 0 to 4) and watershed areas (scored from 0 to 5) on T1w, T2w, and DWI images. The final score represents the extent of damage detected (15, 32). Weeke et al. introduced a score tested on two international cohorts: the scored regions included gray matter, white matter/cortex, and cerebellum. They found that the gray matter subscore was the best predictor of outcomes at 18–24 months and school age (27, 72). Finally, Trivedi et al. scored signal abnormality on T1w, T2w, and DWI sequences in five regions: MRI injury grades were associated with outcomes in the cognitive, motor, and language domains of the Bayley-III scale (73). MRI scoring systems are mostly time-consuming but are generally easily reproducible, with Weeke and Trivedi scores showing some of the highest inter-rater reliability (74).

Overall, higher MRI scores correlate with poorer clinical outcomes, with less extensive brain injury observed in treated neonates (15, 67, 75).

However, certain limitations in outcome prediction should be acknowledged. Kühne et al. found that brain MRI during the first week of life could predict severe neurocognitive outcomes at 5 years of age, as a Weeke score ≥10 is associated with poor outcomes, but modest MRI alterations were weakly predictors of moderate or mild neurodevelopmental impairment (76). Similar results have been reported by the HEAL trial, which demonstrated that major MRI abnormalities at 4–6 DOL predicted severe impairment at 2 years of age, whereas no significant differences in neurodevelopmental outcomes were observed among infants with normal, mild, or moderate MRI findings (77). In addition, some studies have demonstrated differences in the strength of correlation between MRI findings and long-term outcomes. In the study by Mastrangelo et al., the presence of moderate or severe early MRI abnormalities after TH showed poor correlation with an unfavorable developmental outcome, indicating that early abnormal brain MRI findings do not necessarily imply an unfavorable prognosis. Finally, the prognostic value of MRI for outcomes at 12 and 24 months was lower than that reported in previous studies (78). These findings underscore the need for quantitative, advanced, and harmonized imaging predictors. Although MRI scores are not routinely included in radiology reports, they can be useful for family counseling and research purposes. Quantification of brain damage using standardized scoring systems will be essential for identifying imaging biomarkers and optimizing patient care, particularly by recognizing high-risk neonates who may benefit from targeted interventional therapies delivered within an appropriate neuroplasticity time window (45).

## 6 Conclusion

The routine implementation of TH for neonatal HIE poses significant challenges to non-specialized staff related to local resources and expertise: successful patient management relies heavily on collaboration between neonatology, nursing, neuroradiology teams, and parental involvement to achieve the cultural change required to standardize the process.
